# P-2085. Is India Falling Apart in the Battle against Superbugs? A Clinical Pharmacist's Comparative Study Incorporating RE-AIM framework on Antimicrobial Stewardship Programs in Low- and Middle-Income Countries (LMICs)

**DOI:** 10.1093/ofid/ofaf695.2249

**Published:** 2026-01-11

**Authors:** Merle Joanna, Rithik Dharan, K Kirthiram

**Affiliations:** Jaya College of Paramedical Sciences, Chennai, Tamil Nadu, India; The Tamil Nadu Dr. MGR Medical University, Chennai, Tamil Nadu, India; Jaya College of Paramedical Sciences, Chennai, Tamil Nadu, India

## Abstract

**Background:**

Antimicrobial resistance (AMR) is a threat, especially in low- and middle-income countries (LMICs). Antimicrobial stewardship programs (AMSPs) are essential in combating AMR, and clinical pharmacists are vital in ensuring the rational use of antibiotics. Some LMICs, like Kenya and Nigeria, have made remarkable progress in implementing pharmacist-driven AMSPs. Despite India’s National Action Plan on AMR, the country faces challenges in incorporating effective antimicrobial stewardship programs (AMSPs). This study compares India’s progress in fighting AMR to other LMICs by RE-AIM framework tool.

**Figure:**
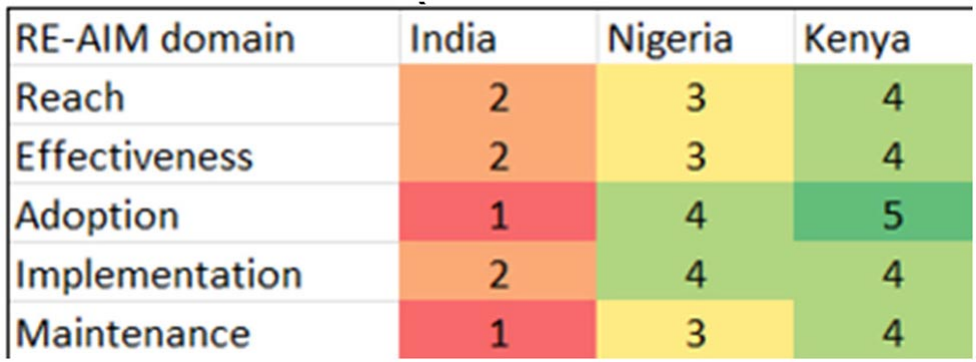
RE-AIM framework comparison across LMIC's

**Figure:**
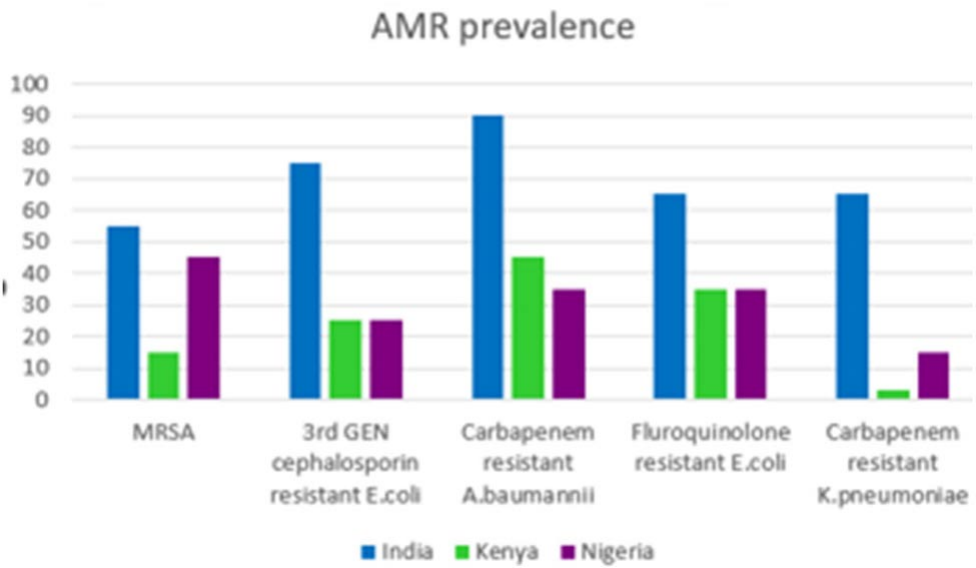
Comparsion of AMR prevalence among LMIC's

**Methods:**

DATA SOURCES: PubMed, The LANCET, World Bank, National antimicrobial policies and national action plan (NAP) by WHO.

DATA EXTRACTION AND DATA SYNTHESIS: AMSP in India, Kenya and Nigeria were evaluated using five RE-AIM domains. A comparative analysis on AMR rate from 1990-2021 and forecast to 2050 among LMICs is also incorporated.

**Figure:**
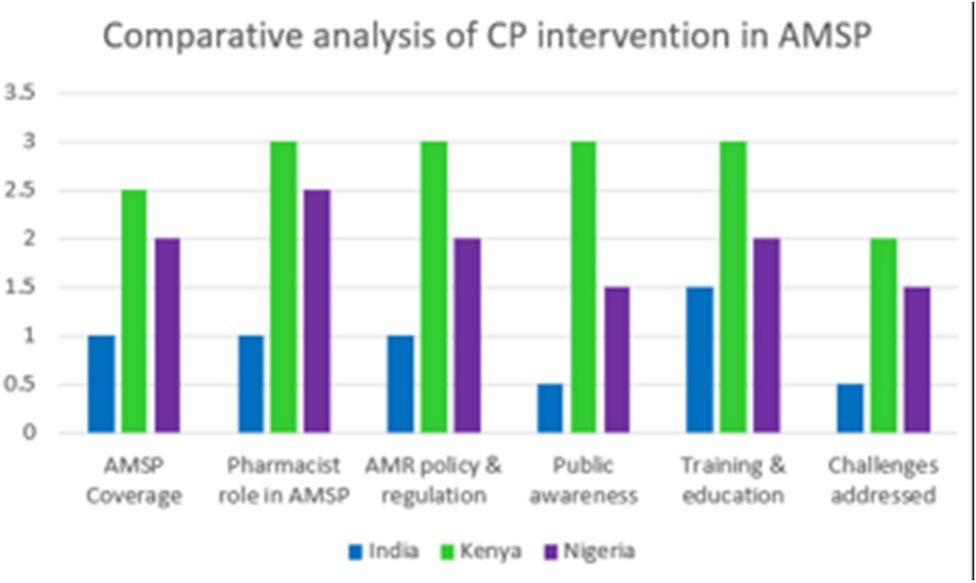
Comparitive analysis of Clinical Pharmacist interventions among LMIC's

**Results:**

A comparative analysis of antimicrobial stewardship programs (ASPs) in low- and middle-income countries (LMICs) discovered significant variations in India's approach to combat antimicrobial resistance (AMR). While Kenya and Nigeria have effectively integrated clinical pharmacists into AMS programs, (fig 1)India continues to face inconsistent policy enforcement, limited AMSP coverage, and overuse of over-the-counter (OTC) antibiotic

**Conclusion:**

India lags in AMSP implementation due to uneven enforcement, a dearth of pharmacist-driven interventions. To combat AMR and improve India's fight against AMR, it is important to strengthen policy enforcement, extend AMSPs beyond tertiary hospitals, integrate pharmacist-driven initiatives, and enhance surveillance systems.

**Disclosures:**

All Authors: No reported disclosures

